# Phylogeography and Phylogenetic Evolution in Tibetan Sheep Based on *MT-CYB* Sequences

**DOI:** 10.3390/ani10071177

**Published:** 2020-07-12

**Authors:** Jianbin Liu, Zengkui Lu, Chao Yuan, Fan Wang, Bohui Yang

**Affiliations:** 1 Lanzhou Institute of Husbandry and Pharmaceutical Sciences, Chinese Academy of Agricultural Sciences, Lanzhou 730050, China; luzengkui@caas.cn (Z.L.); yuanchao@caas.cn (C.Y.); 2 Sheep Breeding Engineering Technology Research Center, Chinese Academy of Agricultural Sciences, Lanzhou 730050, China; 3 China Agricultural Veterinarian Biology Science and Technology Co. Ltd., Lanzhou 730046, China; wangfaneasy@126.com

**Keywords:** Tibetan sheep, *MT-CYB*, phylogeography, maternal lineage

## Abstract

**Simple Summary:**

The molecular and population genetic evidence of the phylogenetic status of the Tibetan sheep (*Ovis aries*) is not well understood, and little is known about this species’ genetic diversity. The aim of the present research was to explore phylogeography and phylogenetic evolution of Tibetan sheep populations, on the basis of mitochondrial DNA (mtDNA) gene *MT-CYB* (1140 base pairs). The results of this study will add information to the Tibetan sheep populations and help enlighten upcoming programs related to conservation of Tibetan sheep living in the Qinghai–Tibetan Plateau.

**Abstract:**

To date, molecular genetics and population studies in Tibetan sheep (*Ovis aries*) have been limited, and little is known about the phylogenetic evolution and phylogeography of Tibetan sheep populations. The aim of the present research was to explore phylogeography and phylogenetic evolution of Tibetan sheep populations, on the basis of mitochondrial DNA (mtDNA) gene *MT-CYB* (1140 base pairs). Our dataset consisted of 641 *MT-CYB* sequences from the same amount of animals belonging to 15 populations of Tibetan sheep living in the Qinghai–Tibetan Plateau, China. Haplotype and nucleotide diversities were 0.748 ± 0.010 and 0.003 ± 0.001, respectively. The analysis of phylogeography revealed the presence of two formerly described haplogroups in 15 populations of Tibetan sheep, however only one haplogroup was present in Awang sheep. Moreover, 641 Tibetan sheep were distributed into a minimum of two clusters by clustering analysis. The 15 Tibetan sheep populations and 19 reference populations of 878 individuals were separated into six main groups based on their substitutions per site, from which we constructed a phylogenetic tree. Minor differences in branching order of various taxa between trees acquired from either gene were observed. This study provides insights on the origins and phylogenetic evolution of populations residing in the Qinghai–Tibetan Plateau, which will aid information of future conservation programs aimed at conserving this valuable genetic resource.

## 1. Introduction

In the Qinghai–Tibetan Plateau, Tibetan sheep have economic, agricultural, cultural, and also religious significance, providing wool, fur and meat to the local people [1]. The Qinghai–Tibetan Plateau has abundant genetic biodiversity of Tibetan sheep of about 2.3 million animals from 16 indigenous sheep populations [2]. These populations are mainly distributed in six regions: the Qinghai Plateau, Gansu Plateau, Tibetan Plateau, and the Sichuan Plateau (all pasture land), and the Yunnan and Guizhou regions (mountainous). Tibetan sheep are highly adapted to their native environment [3]. They are also regarded as important genetic resources and one of the most essential parts of animal husbandry. However, Tibetan sheep have suffered from a serious decline in the numbers of each population for the past 10 years [2]. The unique climate and landforms of the Qinghai–Tibetan Plateau provide Tibetan nomads with a distinct livelihood due to the high-altitude and snow-covered mountains [4]. Traffic from other parts of China is blocked; thus, the Tibetan sheep are rarely influenced by external populations. These populations may now be on the verge of extinction and may ultimately be lost, given the rapid destruction of their ecological environment, the continuing introduction of modern commercial Tibetan sheep populations, and the ongoing lack of effective conservation methods. Tibetan sheep have a special place in the cultural, religious and social life of their herders. The remains of fossils point out that the history of domestic Tibetan sheep to their wild ancestors can be traced back to the Pleistocene period [5]. Archeological evidence showed that yak domestication was performed by the ancient Qiang people of Northern Tibet about 5000 years ago [6]. According to temporal scale, animals native to the Qinghai–Tibetan Plateau are regarded as an ideal model to study adaptation to cold and hypoxia, owing to their adaptions to high altitude hypoxia. Tibetan sheep are sequestered from sheep located in other areas of China, due to their lack of movements to other areas of China or neighbouring countries (Nepal, Pakistan, India, etc). The ecological degradation, environmental changes, illegal commerce, and lack of animal conservation may result in the extinction of Tibetan sheep [7]. To date, phylogenetic relationship, genetic diversity and maternal pedigree of populations in the Qinghai–Tibetan Plateau stay unclear and disputed [2].

The *MT-CYB* gene is one of the genes that is coded by mtDNA, and its gene product plays a key role in the transfer of electrons through the respiration chain [8]. The *MT-CYB* gene has a moderate mutation rate (16.1%) of bases and a clear evolutionary pattern making it well suited for phylogenetic investigations at intra-and inter-specific levels [9]. Current progress in genetics and the usage of mtDNA to distinguish genetic diversity of sheep have provided insights regarding the history of sheep domestication and their human-driven patterns of global migration [10]. Research conducted on the control region fragment and the mitochondrial *MT-CYB* gene in modern groups of sheep with vast geographical origins, has described diverse patterns of genetic diversity in domestic sheep based on five haplogroups [11,12]. Genetic analysis revealed that Haplogroups A and B reside in domestic sheep from every geographic region. Haplogroup A is chiefly indicated in Asian populations [13], however, haplogroup B has a high rate of occurrence in European and Asian populations. In contrast, haplogroup C is rarer, and only a small number of cases in Asia (inside the Fertile Crescent) and Europe (inside the Caucasus and Iberian Peninsula) have been identified [14,15]. Haplogroups D and E were identified more recently and, to date, are the least represented of the five populations; sheep having these haplogroups have so far only been found in the Caucasus and Turkey [12,15]. Current phylogenetic research on the whole mitochondrial genomes of 10 domestic and 6 wild sheep (to examine the genetic link between domestic and wild sheep) confirmed that domestic sheep can be classified according to the five haplogroups; A, B, C, D, and E [16].

The present study investigated *MT-CYB* gene variability in Tibetan sheep indigenous to the Qinghai–Tibetan Plateau, China. A total of 15 Tibetan sheep populations were included in population genetics and phylogenetic analyses of the *MT-CYB* gene. By providing insights into the genetic diversity, maternal pedigree and phylogenetic evolution of Tibetan sheep, our results are relevant to future efforts for conservation and improved management of their genetic diversity.

## 2. Materials and Methods

### 2.1. Sample Collection

All experimental procedures and protocols were approved by the Institutional Animal Care and Use Committee of the Lanzhou Institute of Husbandry and Pharmaceutical Science of the Chinese Academy of Agricultural Sciences (Approval No. NKMYD201805; dated: 18 October 2018). Blood samples from 641 Tibetan sheep were collected. These animals belonged to 15 Tibetan sheep populations that are distributed through Qinghai Province, Gansu Province, and the Tibet Autonomous Region. The genetic characteristics of these Tibetan sheep populations were analyzed in order to determine the phylogeny and phylogeography of the populations. The examined populations were comprised of the following numbers and analogous population types: 8 Duoma Tibetan sheep (DM), 5 Awang Tibetan sheep (AW), 33 Huoba Tibetan sheep (HB), 10 Langkazi Tibetan sheep (LKZ), 77 Gangba Tibetan sheep (GB), 60 Zashijia Tibetan sheep (ZSJ), 46 Jiangzi Tibetan sheep (JZ), 43 Qinghai Oula sheep (QH), 46 Qilian White Tibetan sheep (QL), 64 Tianjun White Tibetan sheep (TJ), 38 Guide Black Fur sheep (GD), 43 Gannan Oula sheep (GN), 62 Qiaoke Tibetan sheep (QK), 58 Minxian Black Fur sheep (MX), and 48 Ganjia Tibetan sheep (GJ) raised in China. Ten milliliter blood samples via the jugular vein were collected from each animal, and 2 mL from each sample were snap frozen in liquid nitrogen and stored at −80 °C for later genomic DNA extraction [17]. Total DNA was extracted from whole blood using the saturated salt method [18]. Isolated DNA was quantified by using a spectrophotometer and concentration was adjusted to 50 ng/μL. The samples details (population code, altitude, longitude, latitude, sample number, accession number, sampling and geographical location) for 15 native Tibetan sheep populations is given in Supplementary Table S1. Altitude, also known as absolute altitude, is the vertical distance from sea level to the collection site, which was measured with a runtastic altimeter PRO and indicated the measurement in meters. 

### 2.2. Data Collection

To attain good coverage of the studied populations, a dataset of 19 reference populations was compiled using 234 submitted sequences comprised of 3 Bashibai sheep (BSB), 3 Henan Big Tail sheep (HN), 4 Akkaraman sheep (AKA), 5 Cypriot mouflon (CM), 12 Madgyal sheep (MD), 12 Morkaraman sheep (MOR), 19 Bannur sheep (BN), 21 Garole sheep (GA), 24 Kolhapuri sheep (KO), 29 Deccani sheep (DE), 49 Bulkhi sheep (BU), 27 Sangamneri sheep (SN), 5 Snow sheep (SS), 8 Tuj sheep (TUJ), 11 Argali, and 2 Musimon *MT-CYB* for the 234 individuals in GenBank. These 19 populations were from six global geographic regions i.e., Turkey, India, Italy, Pakistan, Germany, and China (Supplementary Table S2). 

Primer pairs used for polymerase chain reaction (PCR) and primers for sequencing were designed on the basis of 5′and 3′ conserved flanking sequences of the complete *MT-CYB* gene via Primer Premier 5.0 software [19], and synthesized by BGI Shenzhen Technology Co., Ltd. (Shenzhen, China). The sequence of reverse primer was 5′-TTGGGTGTTGATAGTGGGGC-3′, and the sequence of forward primer was 5′-TGAAGAAAACCCCACAAAACCT-3′. PCR was performed by using a thermal cycler (Mastercycler gradient; Eppendorf, Germany) with a total reaction volume of about 30 µL, having 2 µL of genomic DNA (10 ng/µL), 3 µL (3 pM) of each primer, 3 µL10 × Ex Taq™ reaction buffer, 2 µL dNTP (2.5 mM), 0.2 µL Taq DNA polymerase (5 U/µL) (TaKaRa, Dalian, China), and 16.8 µL double-distilled H_2_O (ddH_2_O). The PCR conditions were as follows: initial denaturation at 94 °C for 5 min, 36 cycles of denaturation for 30 s at 94 °C, annealing for 30 s at 56 °C, and extension for 1.5 min at 72 °C. A final 10-min extension was performed at 72 °C. The amplified products were subsequently stored at 12 °C until use.

The amplified fragments of the *MT-CYB* gene were purified using a PCR gel extraction kit from Sangon Biotech Co., Ltd. (Shanghai, China) and directly sequenced using BigDye^®^ Terminator v3.1 cycle sequencing ready reaction mix (Applied Biosystems, Darmstadt, Germany) in an automatic sequencer (ABI-PRISM 3730 genetic analyzer, Applied Biosystems, Foster City, CA, USA) and sequenced fragments of the *MT-CYB* gene were assembled by MITObin software (V 1.7) [20]. PCR for sequencing was done in an automatic sequencer with a total reaction volume of about 5 µL having 3 µL genomic DNA (10 ng/µL), 1µL (3 pM) of each sequencing primer, 0.5 µL BigDye, and 0.5 µL ddH_2_O. The sequencing conditions were as follows: initial denaturation at 95 °C for 2 min, 25 cycles of denaturation for 10 s at 95 °C, and annealing for 10 s at 51 °C. The final extension step was followed by a 190 s extension at 60 °C. The PCR sequencing products were subsequently stored at 12 °C until use.

### 2.3. Data Analysis

The sequences were organized using Clustal Omega tool for multiple comparison and were aligned using BLAST and ClustalW 1.2.0 [21,22]. These results were compared with three outgroup sequences of wild yak, domestic yak and cattle, obtained from GenBank (accessions given in Supplementary Table S2). The diversity parameters such as haplotype diversity and nucleotide diversity were assessed using DnaSP (Sequence Polymorphism Software) 5.10.01 [23]. The genetic differentiation coefficient (*G_ST_*), and Wright’s subpopulation within total population F-statistic (*F_ST_*), were assessed using Arlequin version 3.5.1.2 [24]. 

The molecular variance (AMOVA) test was estimated using Arlequin version 3.5.1.2 [24]. Four groups were established to identify differences between geographic regions using the AMOVA program. The phylogenetic tree was obtained alternately using the Bayesian inference (BI), and maximum likelihood (ML), Maximum parsimony (MP) and Neighbor Joining (NJ) methods, with the HKY + G model [25], respectively. The posterior probabilities for the branches and bootstrap values were also calculated. DnaSP v5 software was used to analyze the haplotype contruction between sites [23]. The phylogeny and molecular evolutionary relationships, ME Haplotype phylogenies and clustering tree, and genetic distance were determined using MEGA version 6.0 [26]. We drew the network and mismatched distribution graphs using a median joining method implemented in NETWORK software (version 4.6.1.2) to determine the haplotype relationships [27].

## 3. Results

### 3.1. Polymorphic Sites and Sequencing Analysis of the MT-CYB Gene

All the sequences were aligned with 1140 comparative site and 132 haplotypes were attained from 641 sequenced animals. Sequences from 641 animals (from 15 Tibetan populations) showed considerable length heteroplasmy (the most abundant of which was 1140 base pairs). In total, 157 variable sites were attained from sequences that included 71 singleton variable sites and 86 parsimony informative variable sites. Out of 157 variable sites, 127 were transitions and 30 were trans-versions, among which 15 sites were both transitions and trans-versions. The observed substitutions caused the transition mutations.

**Figure 1:**
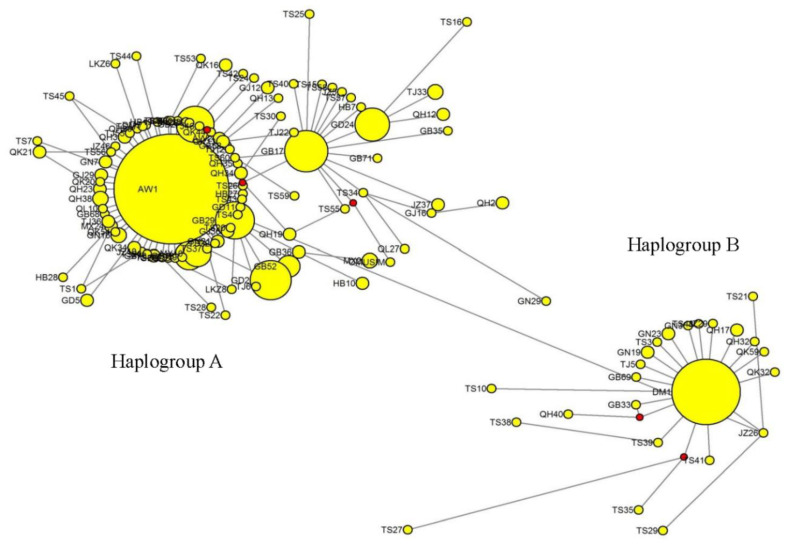
Median-joining networks for the *MT-CYB* in 641 individual from 15 Tibetan sheep populations. The haplogroup A consisted of 560 individuals and 175 haplotypes; Haplogroup B consisted of 81 individuals and 39 haplotypes.

Whole haplotypes’ nucleotide compositions were: 31.472% A, 27.174% T, 28.441% C, and 12.913% G, 58.646% A + T and 41.354% G + C, while A + T were substantially more common than the G + C haplotype, displaying an AT bias. The ME phylogenetic tree shows that the 132 haplotypes of the 15 Tibetan sheep populations fall into two primarily distinct clusters: haplogroup A and haplogroup B (Supplementary Figure S1). The main haplogroup A was comprised of 560 animals and 175 haplotypes; the second main haplogroup B contained 81 animals and 39 haplotypes (Figure 1, Supplementary Figure S1). The number of different haplotypes, individual animals, and haplotype frequencies (i.e., rate of occurrence for a specific haplotype) for each population of Tibetan sheep ranged from 2 to 60, 0 to 67, and 0 to 1.000, respectively. In total, 132 haplotypes were identified in the present study and diversity of haplotype and nucleotide were estimated at 0.748 ± 0.010 and 0.003 ± 0.001, respectively. These data show that genetic diversity is high for the 15 target populations of Tibetan sheep. The nucleotide diversity value of the Zashijia Tibetan sheep (ZSJ) population (0.006 ± 0.001) was higher than for the other 14 populations (indicating greater genetic diversity). Likewise, haplotype diversity was maximum in ZSJ population (1.000 ± 0.000) and minimum in the Duoma Tibetan sheep (DM) population (0.464 ± 0.040) (Table 1).

### 3.2. Genetic Distance and Genetic Differentiation

Data are shown in Supplementary Table S3 for genetic distance and genetic differentiation in 15 populations of Tibetan sheep. Genetic distances reached from 0.004 to 0.048 inside the populations (along the diagonal), and from 0.004 to 0.043 between populations (above the diagonal). Within-population genetic distances were minimal in the Awang (AW) population, and maximal in the ZSJ population. Between-population genetic distances were greatest for the JZ and ZSJ populations, and lowest for the DM and AW populations. 

We calculated *F_ST_* to study the genetic differentiation among 15 populations of Tibetan sheep. Estimates for pair-wise *F_ST_* values (below the diagonal), calculated to study the genetic differentiation among the target Tibetan sheep populations, were from 0 to 0.118, *p*-value from 0 to 0.072, respectively (Supplementary Table S3). Of all the populations, AW and ZSJ populations were the largest pairwise *F_ST_* value (*F_ST_* = 0.118, *p*-value = 0.052). Moreover, all *F_ST_* values were less than 0.15 (range: 0 to 0.118), showing that significant genetic differentiation has little differentiation among populations. Supplementary Table S3 presents the genetic distance between and within the fifteen Tibetan sheep populations. The genetic distance values ranged from 0.004 to 0.048 within the population diagonals, and the genetic distance values ranged from 0.009 to 0.040 among populations above the diagonals. Among the Tibetan sheep populations, the genetic distance within populations reached a maximum value in Zashijia Tibetan sheep and a minimum value in Awang sheep. Different populations varied in their inter-relatedness, as shown by the distribution pattern of pairwise *F_ST_* values (Supplementary Table S3). Our analyses showed that gene divergence among populations is very low. Therefore, the variations observed among the 15 populations of Tibetan sheep included in the present study cannot be explained by inter-population genetic differences.

### 3.3. Genetic Distance and Altitude

**Figure 2:**
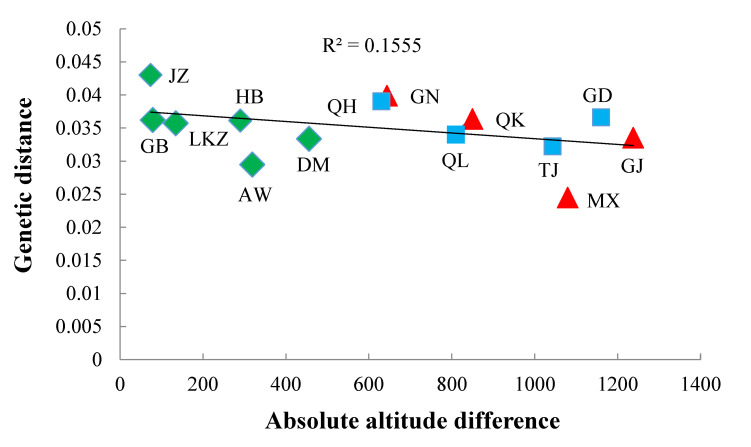
The Genetic distance and absolute difference between altitudes for the population of Zashijia Tibetan sheep (ZSJ). The red triangle, blue square and green rhombus indicate that the respective samples originated from Gansu, Qinghai and Tibet province.

We assess if genetic distances between populations can be described by absolute differences between altitudes for 15 populations of Tibetan sheep. The genetic distance between the main population of ZSJ and each of the remaining populations was plotted in Figure 2 as a function of absolute difference in altitudes. Genetic distance inclined to reduce with the absolute difference in altitudes as estimated by Pearson correlation coefficient (r = −0.3943, two-tailed *p* = 0.163 and square root of 0.1555 indicated in the Figure 2). This tendency is observed in 13 out of 15 populations of sheep, however no statistical significance (*p* < 0.05) was recorded (see Table 2). It is strongest (most negative) for populations of high altitude (DM, AW, HB, LKZ, GB, JZ, ZSJ, QH, QL, TJ, GN, QK, and MX), and weakest (most positive) for low altitude populations (GD and GJ). The relationship between altitude and Pearson correlation coefficients achieved between genetic distances and absolute differences in altitudes (Table 2) has r = −0.42 and one tailed *p* = 0.0028.

### 3.4. Genetic Differentiation and Altitude

**Figure 3:**
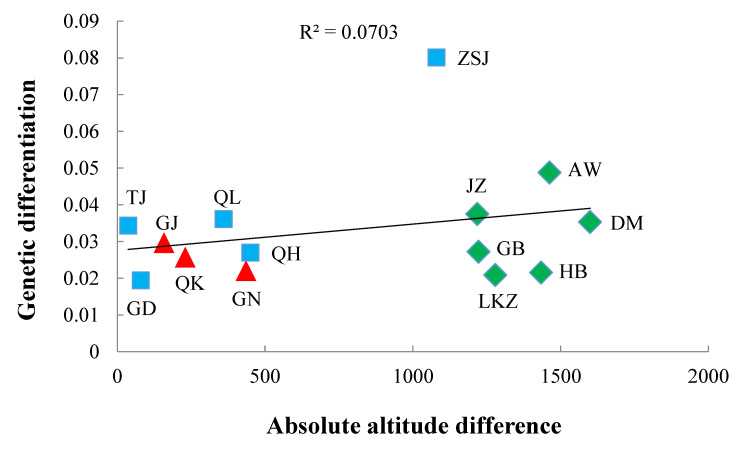
The genetic differentiation and absolute difference between altitudes for the population of Minxian Black Fur sheep (MX). The red triangle, blue square and green rhombus indicate that the respective samples originated from Gansu, Qinghai and Tibet province.

We assessed if genetic differentiation between populations can be clarified by altitude. The genetic differentiation between the main population MX and each of the remaining populations were plotted in Figure 3 as a function of absolute value of difference between their altitudes. Genetic differentiation inclined to reduce with altitude (r = 0.2625, one tailed *p* = 0.018) (coefficient of determination R^2^ of 0.0703 showed in Figure 3). Analyses in Figure 3 displayed that genetic differentiation increases with absolute differences in altitude, specifically for ten populations i.e., ZSJ*, AW, QK, GJ, TJ, MX, QH, GN, GD, and QL (significance at *p* < 0.05 shown by *) and decreases for the five populations i.e., GB, JZ, LKZ, HB and DM. 

This relationship between genetic differentiation and absolute difference between altitudes is more positive for low altitude populations and more negative for high altitude populations. This relationship between altitude and Pearson correlation coefficients attained between genetic differentiations and absolute differences in altitudes (Table 2) has r = −0.13 and two tailed *p* = 0.0011.

### 3.5. Phylogenetic Relationship

**Figure 4:**
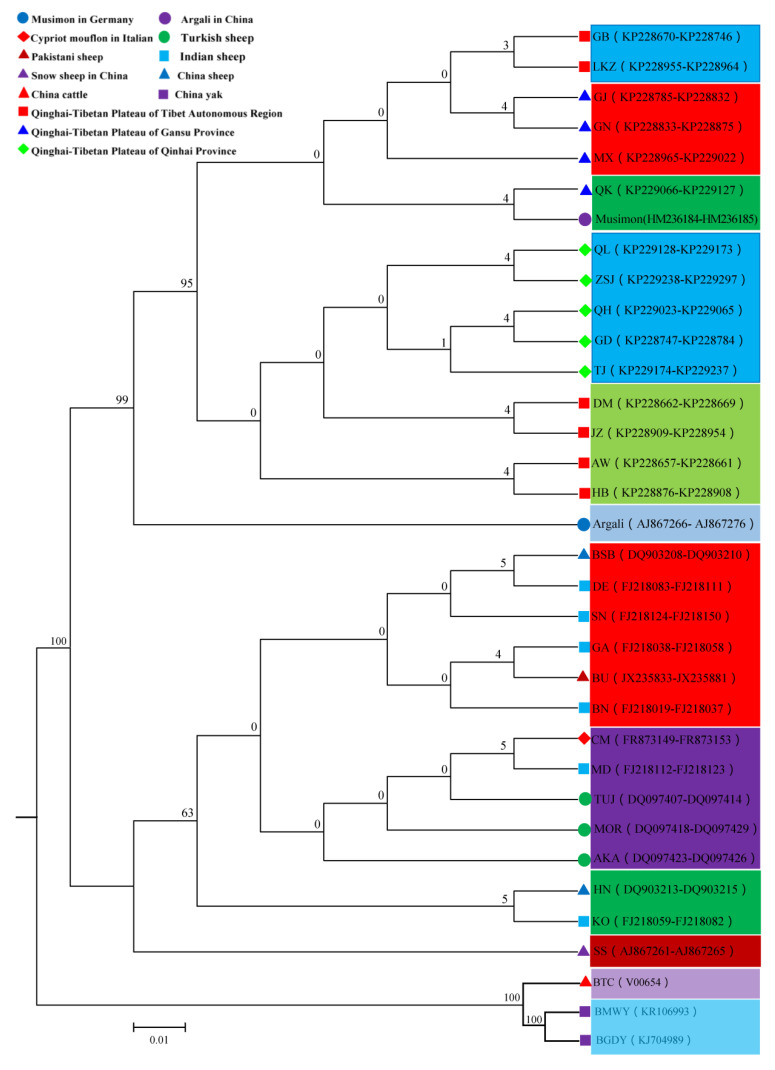
Neighbor joining Phylogenetic Tree of 34 Populations on the basis of 878 Sequences of *MT-CYB*. The distances were calculated by using the Maximum Composite Likelihood method and units are the number of base substitutions per site.

To enhance our information about phylogenetic relationships among 34 populations, we constructed a phylogenetic tree using neighbor-joining (NJ) based on complete *MT-CYB* gene sequences of 875 animals from 15 populations of Tibetan sheep and 19 reference populations (Supplementary Tables S1 and S2). The neighbor-joining tree based on the complete *MT-CYB* gene of target Tibetan sheep and reference populations is reported in Figure 4. The 34 populations were distributed into eight sub clusters. The first cluster was comprised of GB, LKZ, GJ, GN, MX, QK and Musimon, which may be the matriarchal origin of the six Tibetan sheep from Musimon. The second cluster was comprised of QL, ZSJ, QH, GD, TJ, DM, JZ, AW, and HB. The third cluster only included Argali. The fourth cluster included BSB, DE, SN, GA, BU, and BN. The fifth cluster included CM, MD, TUJ, MOR, and AKA. The sixth cluster included HN and KO. The seventh cluster only included SS. The eighth cluster uncluded BMWY, BGDY, and BTC (outgroup). 

The ME phylogenetic tree shows that the 132 haplotypes of Tibetan sheep populations fall into two primarily distinct clusters: haplogroup A and haplogroup B (Supplementary Figure S1). Haplogroup A consisted of 560 individuals and 175 haplotypes; Haplogroup B consisted of 81 individuals and 39 haplotypes (Figure 1). Of the 132 haplotypes, there was no common haplotype identified in all of the Tibetan sheep populations; 82 haplotypes were shared, and 50 haplotypes were singletons, including 60 in Zashijia Tibetan sheep, 21 in Qinghai Oula sheep, 18 in Tianjun White Tibetan sheep, and 17 in Gangba Tibetan sheep. The leading haplotype (Haplotype 1) was found in 318 individuals. The next most common haplotype was Haplotype 3, composed of 55 individuals, followed by Haplotype 6 and Haplotype 21, respectively consisting of 22 and 20 individuals, and the remaining haplotypes were composed of one to 18 individuals. Haplotype 1 was composed of 15 of the Tibetan sheep populations and showed close clustering. AMOVA revealed that between-population and within-population genetic variation was 8.89% and 91.11%, respectively (*p* < 0.001) (Table 3). The *F_ST_* value was 0.089, indicating that 8.9% of the total genetic variation was because of between-population differences and the remaining 91.1% was due to within-population differences (i.e., differences among animals of the same population).

### 3.6. Population Expansions

**Figure 5:**
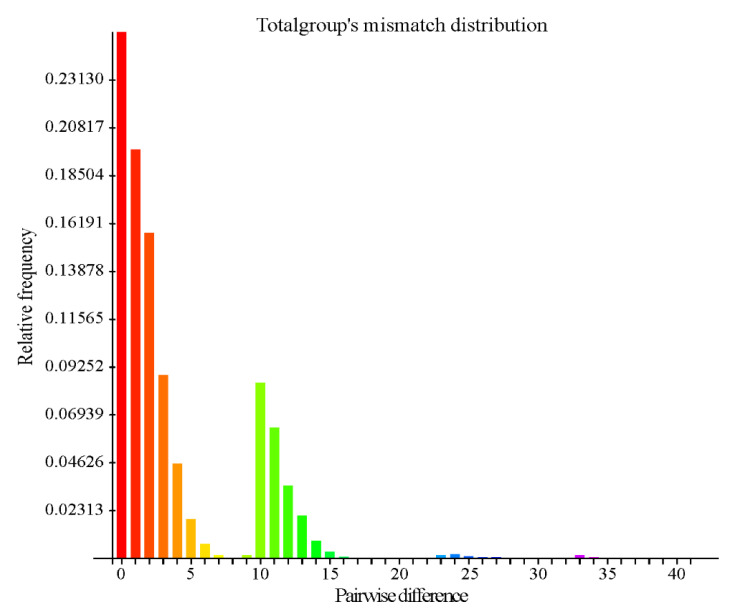
The mismatch distribution of the complete dataset of the *MT-CYB* types of Tibetan sheep of the four lineages on the Qinghai–Tibetan Plateau areas showed that there were two major peaks, with maximum values at 0 and 10 pairwise differences, and two smaller peaks at 24 and 33 pairwise differences.

The mismatch distribution analysis of the complete dataset (haplogroups A, B, and 15 Tibetan sheep populations of *MT-CYB*) is shown in Figure 5. The charts of the mismatch distribution for the samples of the 15 Tibetan sheep populations and the total samples were multimodal. The mismatch distribution of the complete dataset showed that there were two major peaks, with maximum values at 0 and 10 pairwise differences, and two smaller peaks at 24 and 33 pairwise differences (Supplementary Figure S2). These results suggest that at least two expansion events occurred during the population demographic history of the Tibetan sheep population. The mismatch distribution analysis revealed a bimodal bell-shaped distribution of pairwise sequence differences in lineages A and B. Mismatch analysis of lineages A and B suggested that a single population expansion event occurred in the demographic history of Tibetan sheep populations. This finding suggests the occurrence of two expansion events in the demographic history of the 15 Tibetan sheep populations. This result is consistent with a demographic model showing two large and sudden expansions, as inferred from the mismatch distribution. 

## 4. Discussion

### 4.1. High MT-CYB Gene Diversity of Tibetan Sheep Populations

The fifteen Tibetan sheep populations in our study showed a high level of haplotype and nucleotide diversity. Although this result is higher than an estimate given in a previous report [28], this is probably due to differences in the lengths of the sequences that were studied. In this research, the complete sequence of the *MT-CYB* was studied, while Zhang et al. only examined 450 bp of the gene [28]. Among 641 sequences, only 157 mutations were identified and nucleotide diversity was estimated at 0.003 ± 0.001. Although consistent with other genetic diversity studies [29,30], estimates for haplotype and nucleotide diversities were lower than in a previous study that examined the MT-CYB [28], indicating relative conservation of the *MT-CYB*. 

Our findings show comparatively greater genetic diversity in 15 populations of Tibetan sheep in comparison to other sheep populations [7,31]. Most of the identified base substitutions were silent (i.e., the encoded amino acid was not changed). In other studies on Chinese sheep breeds, genetic diversity was found to be lower [32]. In contrast, our data (based on analysis of the *MT-CYB*) show higher genetic diversity for 15 populations of Tibetan sheep in the Qinghai–Tibetan Plateau (641 total animals). The haplotype diversity was calculated separately for each Tibetan sheep population and was estimated to be 0.992 ± 0.010, which was based on mtDNA D-Loop sequences with 15 Tibetan sheep populations of 636 individuals [24]. We therefore conclude that genetic diversity, compared with other populations, is high for Tibetan sheep populations. However, according to a previous study for analysis of conservation units, a sample size of 59 animals is needed to reject the hypothesis that animals with hidden character positions occur in populations. The sample size required to reject the presence of hidden states (at a hypothetical frequency e.g., 0.05) is slightly smaller (56 for a population of 500) when sampling from a limited population. Thus, the large sample size of Tibetan sheep populations in the present study was appropriate to reliably determine genetic diversity. For LKZ, LZ, HB, GD, QH, TJ, QK, GJ, GB, and GN Tibetan sheep populations (which have broad geographic distributions), detection of high genetic diversity is dependent on a sufficiently large sample size and a wide collection area. Thus, a larger sample size may have revealed even greater genetic diversity in these 15 populations of Tibetan sheep, and further studies may be warranted to explore this possibility. However, the sample size of DM, AW, and LKZ was small. 

The Tibetan sheep populations (which have been characterized as the rarest) encountered a genetic bottleneck during the 20th century [33]. Consistent with the findings of Tapio et al., outcomes of the positive neutrality tests were considerably varied among 15 populations of Tibetan sheep, proposing an earlier decrease in *MT-CYB* diversity [33]. Among factors that may have influenced *MT-CYB* diversity are an increased mutation rate (for this gene), mingling of populations from various geographical locations, overlapping generations, natural selection choosing heterozygosis, maternal effects of numerous wild ancestors and subdivision complemented by genetic drift [1].

### 4.2. Maternal Pedigrees of Tibetan Sheep Populations

Sequencing data of *MT-CYB* (1140 base pairs) revealed that 15 Tibetan sheep populations cluster into our sub-clusters. The rich mtDNA diversity in Tibetan sheep populations suggests a non-widespread origin for maternal lineages [34,35]. Moreover, the present study revealed a significant bio-geographic association for the Tibetan sheep populations, with the first cluster found to contain animals belonging to Gansu (GJ, GN, MX and QK) and Tibet (GB and LKZ) 6 Tibetan sheep populations, which revealed that the maternal pedigrees are Musimon. In addition, the first cluster has been proposed as being particularly representative of the thoroughbred Gansu and Tibetan sheep population. The results showed that the matrilineal origin of the first cluster of 6 Tibetan sheep populations is closest to that of Musimon. In the present study, the second cluster was found to contain animals belonging to Qinghai (QL, ZSJ, QH, GD, and TJ) and Tibet (DM, JZ, AW, and HB) 9 populations of Tibetan sheep. The first and second clusters were clustered with the Argali, and the results showed that maternal pedigree of 15 populations of Tibetan sheep was closest to that of Argali. The fourth cluster was found to contain animals belonging to Indian (DE, SN, GA, and BN), Pakistani (BU), and the Chinese population of Xinjiang of the 1 sheep population (BSB). The fifth cluster was found to contain animals belonging to Italian (CM), Indian (MD), and Turkish (TUJ, MOR, and AKA) members of the 5 sheep populations. The sixth cluster was found to contain animals belonging to Indian (KO) and Chinese (HN) members of the 2 sheep populations. 

The origins of the domestic sheep have been subject to debate. On the basis of studies on the D-loop region of mtDNA in domestic sheep, Hiendleder et al. identified that two maternal lineages are apparent [36]. NJ phylogenetic tree analyses (in the present study) revealed four maternal lineages for populations of Tibetan sheep in the Qinghai–Tibetan Plateau, China. Of these maternal lineages, the first cluster predominated over the second cluster, third cluster, and fourth cluster. These results are consistent with other work done on populations of domestic sheep present in China [7,13]. Previous studies on sheep populations in China [10,30,37] and other countries [15,38] identified three mtDNA maternal lineages (first, second, and third). Lately, in domestic sheep, a novel maternal lineage (fourth) was identified [12]. The results of the present study on populations of Tibetan sheep of the Qinghai–Tibetan Plateau region of China additionally support the hypothesis that Chinese domestic sheep have multiple maternal pedigrees. 

### 4.3. Genetic Differentiation of Tibetan Sheep Populations

*MT-CYB* has been used to study phylogenetic relationships at intra-and inter-specific levels, and has been used in gene flow studies [39,40]. It is mostly accepted that animals encounter a genetic bottleneck effect after their domestication. The value of *F_ST_* characterizes genetic differentiation levels inside a certain population. Differentiation is considered “low” with a value of 0.05, “moderate” with values from 0.05 to 0.25, and “substantial” with values > 0.25 [41]. The present study displayed a significant genetic within-population variation in Tibetan sheep of the Qinghai–Tibetan Plateau, China by using AMOVA. Gene flow (*N_m_*) (also known as gene migration), refers to the transfer of alleles from one population to another. *N_m_* values for haplotype > 1 and for sequence < 1 indicate poor gene exchange, resulting in a situation whereby genetic drift will cause substantial differentiation in local populations [34,42]. In the present study, low *F_ST_* values indicate that genetic variations mainly resulted from independent evolution of the isolated populations, with substantial differentiation in local populations caused by genetic drift [43]. Toward understanding the reason for these variations, lower effective population sizes are likely to have been an important factor. For example, LKZ, GB, GD, GN, QK, GJ, and QL populations living in valleys and gulfs hold limited migration ability; thus, their effective population size, comparative to other populations of Tibetan sheep, is lower. The substitutions of nucleotides have a higher probability of reaching fixation, as the size of effective population decreases [44]. Thus, an important part might have played by known paleo-geographic events in the speciation of Tibetan sheep.

### 4.4. Genetic Relationship among Tibetan Sheep Populations

The present study exhibited that 15 populations of Tibetan sheep belonging to the Qinghai–Tibetan Plateau, China are grouped into two haplogroups: 560 animals belonged to haplogroup A, and 81 to haplogroup B. These genetic groupings are highly consistent with conventional schemes of classification and the findings of other investigations [7,13]. Genetic variation among 15 populations of Tibetan sheep could be attributable to several major factors, including geographic segregation, differences in environmental situations and habitat, natural selection and history of breeding. As a moveable source of wool and food, commercial trade and wide displacement of sheep alongside transport routes established by humans may have promoted particular patterns of genetic exchange, resulting in the emergence of different breeds [45]. Other genetic methods i.e., degree method and clustering method for phylogenetic relationship, have showed three maternal lineages of origin for indigenous Chinese sheep [37,46].

### 4.5. Phylogenetic Analysis of Tibetan Sheep Populations

A greater resolution among wild sheep and the main lineages of domestic sheep was displayed by phylogenetic analyses of whole mitogenomes [10]. *MT-CYB* from the whole mitogenomes created similar phylogenies with completely resolved phylogenetic relationships of wild sheep, however, they were unable to describe phylogenetic relationships among main lineages of domestic sheep. Our findings propose that partial fragments of whole mitogenomes would be troublesome in constructing phylogenetic inferences related to domestic sheep. This issue appears because of the location of diagnostic substitutions elsewhere in the mitogenome [10]. So, diagnostic substitutions for species and lineages displayed [10] in this study can act as a significant reservoir for maternal genetic differentiation between wild and domestic sheep and also between lineages inside domestic sheep. Moreover, they might be helpful to address definite disputes discussed in the future.

## 5. Conclusions

Tibetan sheep populations are abundant in China, and there is substantial mtDNA genetic diversity in sheep residing in the Qinghai–Tibetan Plateau. The present study, based on large-scale analysis of the mtDNA *MT-CYB* in 15 populations of Tibetan sheep, provides proof of two maternal lineages with greater genetic diversity. Analysis of phylogeny exhibited that two formerly described maternal lineages could be recognized in 641 tested animals of 15 populations of Tibetan sheep. The origin of the maternal line may be Musimon and Argali.

## Figures and Tables

**Table 1:** Genetic diversity indices for 15 populations of indigenous Tibetan sheep.

Population	Nucleotide Diversity	Haplotype Diversity	No. of Animals	No. of Haplotypes	Haplogroup A		Haplogroup B	
					No. of Animals	Frequency or No. of Haplotypes	No. of Animals	Frequency or No. of Haplotypes
DM	0.003 ± 0.001	0.464 ± 0.040	8	3	7	0.667/2	1	0.333/1
AW	0.001 ± 0.000	0.600 ± 0.031	5	2	5	1.000/2	0	0
HB	0.003 ± 0.001	0.691 ± 0.007	33	10	29	0.900/9	4	0.100/1
LKZ	0.003 ± 0.001	0.778 ± 0.019	10	6	9	0.833/5	1	0.167/1
GB	0.003 ± 0.001	0.714 ± 0.003	77	17	67	0.824/14	10	0.177/3
JZ	0.005 ± 0.001	0.719 ± 0.003	46	11	33	0.727/8	13	0.273/3
ZSJ	0.006 ± 0.001	1.000 ± 0.000	60	60	48	0.800/48	12	0.200/12
QH	0.004 ± 0.001	0.860 ± 0.003	43	21	38	0.810/17	5	0.191/4
QL	0.002 ± 0.001	0.536 ± 0.007	46	9	41	0.889/8	5	0.111/1
TJ	0.002 ± 0.001	0.570 ± 0.006	64	18	61	0.833/15	3	0.167/3
GD	0.003 ± 0.001	0.615 ± 0.007	38	9	32	0.889/8	6	0.111/1
GN	0.004 ± 0.001	0.704 ± 0.006	43	13	34	0.692/9	9	0.308/4
QK	0.003 ± 0.001	0.690 ± 0.004	62	16	54	0.813/13	8	0.188/3
MX	0.002 ± 0.001	0.682 ± 0.002	58	9	57	0.889/8	1	0.111/1
GJ	0.002 ± 0.001	0.701 ± 0.004	48	10	45	0.900/9	3	0.100/1
Total	0.003 ± 0.001	0.748 ± 0.010	641	214	560	0.818/175	81	0.182/39

Total haplotypes, 132; shared haplotypes, 82 haplotypes.

**Table 2:** The altitude and Pearson correlation coefficients between absolute differences in altitude and each population.

Population	Altitude, m	Genetic Dist, r	Two-Tailed *p* Value	Genetic Diff, r	Two-Tailed *p* Value
DM	4780	−0.242	0.404	−0.039	0.894
AW	4643	−0.419	0.136	0.296	0.305
HB	4614	−0.234	0.422	−0.371	0.192
LKZ	4459	−0.361	0.205	−0.051	0.862
GB	4403	−0.483	0.134	−0.390	0.168
JZ	4398	−0.271	0.349	−0.176	0.546
ZSJ	4260	−0.394	0.163	0.513	0.061
QH	3630	−0.153	0.602	0.193	0.509
QL	3540	−0.168	0.566	0.141	0.630
TJ	3217	−0.094	0.749	0.222	0.446
GD	3100	0.150	0.608	0.170	0.560
GN	3616	−0.119	0.685	0.184	0.529
QK	3410	−0.020	0.945	0.265	0.360
MX	3180	−0.269	0.353	0.265	0.360
GJ	3022	0.098	0.739	0.245	0.398

**Table 3:** Hierarchical molecular variance (AMOVA) of the MT-CYB in 15 indigenous Tibetan sheep populations.

Source of Variation	d.f.	Sum of Squares	Variance of Components	Percentage of Variation	*p* Value
Between-population	14	130.85	0.17	8.89	<0.0001
Within-population	627	1096.78	1.75	91.11	<0.0001
Total	641	1227.63	1.92		
*F_ST_*	0.09				

d.f. = degrees of freedom, *F_ST_* = Wright’s subpopulation within total population F-statistic.

## Supplementary Materials

The following are available online at https://www.mdpi.com/2076-2615/10/7/1177/s1, Table S1: Sampling information for the 15 indigenous Tibetan sheep populations. Table S2: Mitochondrial genomes of the 19 populations of 237 sequences included in phylogenetic analyses of this study. Table S3: Estimates of pairwise FST values and p-value (below the diagonals) and genetic distance (above the diagonals) between and within (diagonal) 15 Tibetan sheep populations. Figure S1: The ME phylogenetic tree of 132 haplotypes. The ME phylogenetic tree show that the 132 haplotypes of Tibetan sheep populations fall into two primarily distinct clusters: haplogroup A and haplogroup B. Haplogroups for individuals defined by the entire haplotypes are shaded in blue (haplogroup A) and red (haplogroup B). Figure S2: The Mismatch Distribution of Complete Dataset of Two Lineages of the 15 Tibetan Sheep Populations. The results were summarized in two lineages of the MT-CYB types of the 15 Tibetan sheep populations on the Qinghai–Tibetan Plateau areas showed that there was at least one demographic expansion.
