# Supplementary material for: Phylogeography and Phylogenetic Evolution in Tibetan Sheep Based on MT-CYB Sequences
Source: Animals (Basel). 2020 Jul 12;10(7):1177. doi: 10.3390/ani10071177 (PMC7401538; doi:10.3390/ani10071177)
Supplement: Supplementary file 1 [file animals-10-01177-s001.zip › animals-776681-supplementary/Supplementary Figure.pdf]

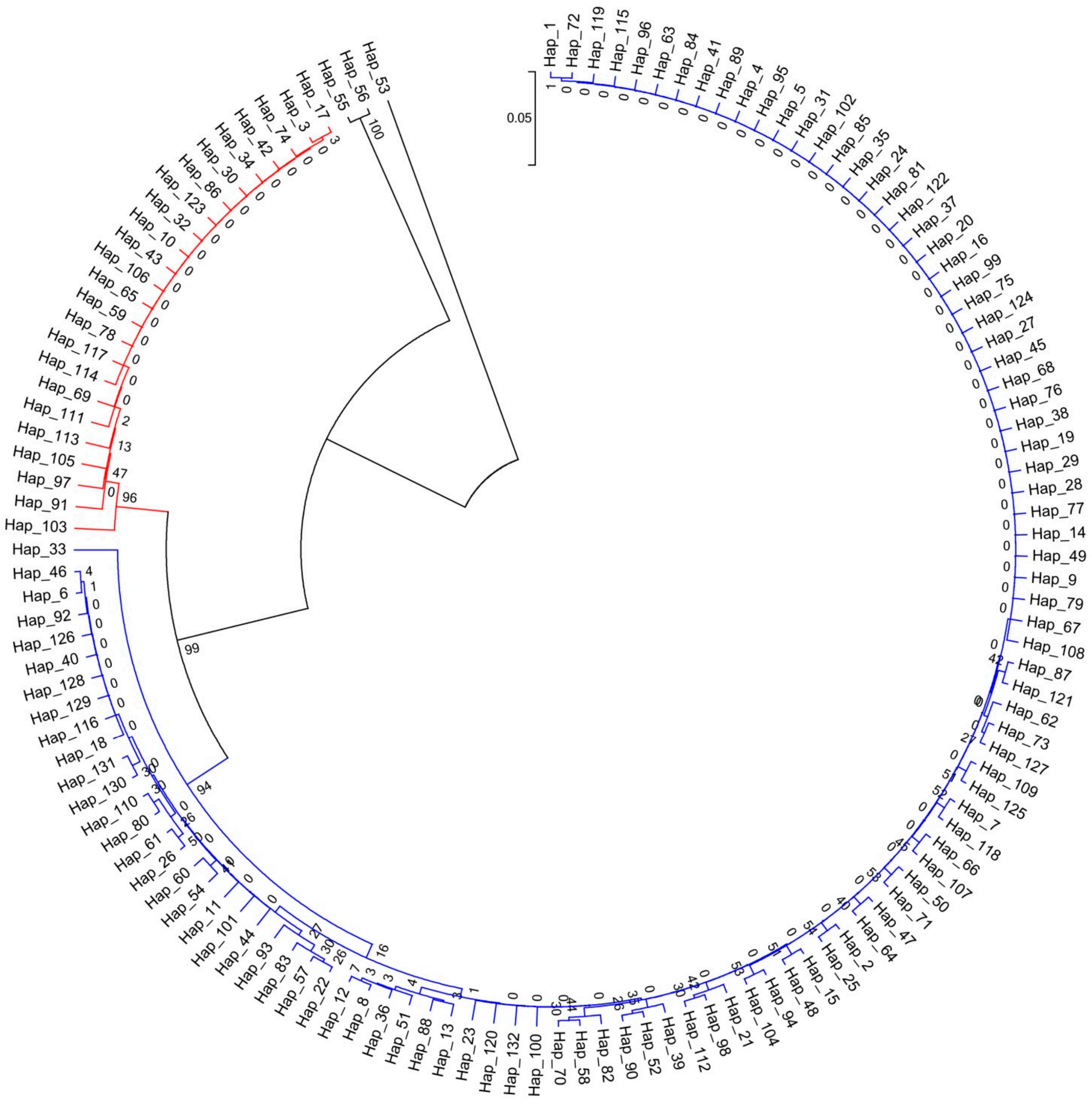

Supplementary Figure S1. The ME phylogenetic tree of 132 haplotypes. The ME phylogenetic tree show that the 132 haplotypes of Tibetan sheep populations fall into two primarily distinct clusters: haplogroup A and haplogroup B. Haplogroups for individuals defined by the entire haplotypes are shaded in blue (haplogroup A) and red (haplogroup B).

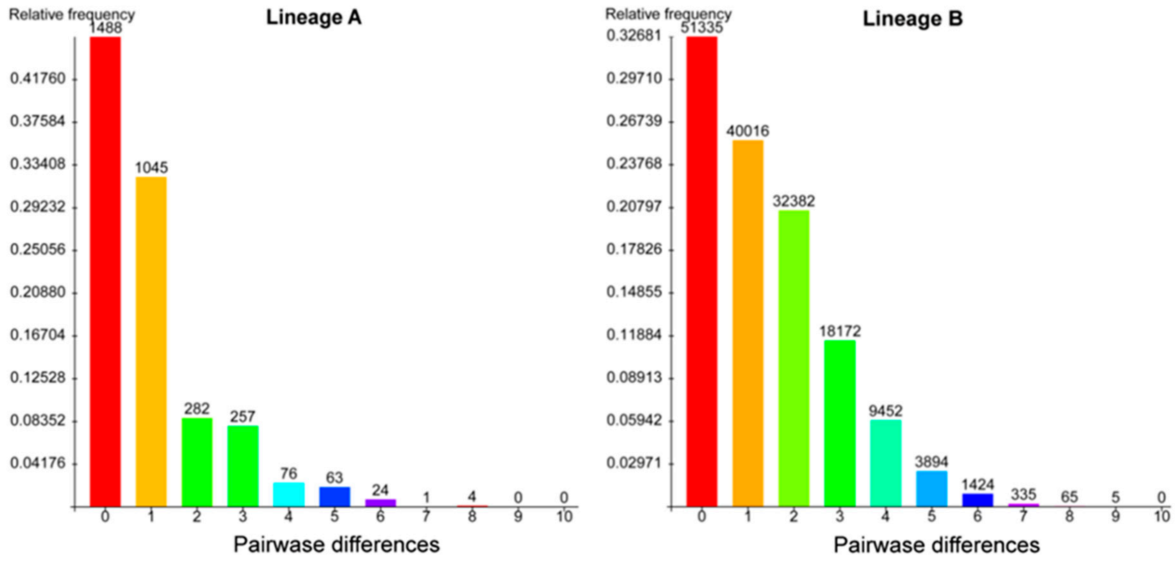

Supplementary Figure S2. The Mismatch Distribution of Complete Dataset of Two Lineages of the 15 Tibetan Sheep Populations. The results were summarized in two lineages of the *MT-CYB* types of the 15 Tibetan sheep populations on the Qinghai-Tibetan Plateau areas showed that there was at least one demographic expansion.
