# Supplementary material for: Phylogeography and Phylogenetic Evolution in Tibetan Sheep Based on MT-CYB Sequences
Source: Animals (Basel). 2020 Jul 12;10(7):1177. doi: 10.3390/ani10071177 (PMC7401538; doi:10.3390/ani10071177)
Supplement: Supplementary file 1 [file animals-10-01177-s001.zip › animals-776681-supplementary/Supplementary Table.pdf]

Supplementary Table S1. Sampling information for the 15 indigenous Tibetan sheep populations.

Population	Population code	Sample number	Altitude (m)	Longitude and latitude	Accession number	Sampling location
Duoma Tibetan sheep	DM	8	4780	N:29°48′609″<br>E:091°36′191″	KP228662-KP228669	Sixth Village, Maqu Town, Anduo County, Naqu Territory of Tibet Autonomous Region
Awang Tibetan sheep	AW	5	4643	N:30°12′101″<br>E:098°63′098″	KP228657-KP228661	Ayi third Village, Awang Town, Gongjue County, Changdou Territory of Tibet Autonomous Region
Huoba Tibetan sheep	HB	33	4614	N:30°13′822″<br>E:083°00′249″	KP228876-KP228908	Rima Village, Huoba Town, Zhongba County, Rikaze Territory of Tibet Autonomous Region
Langkazi Tibetan sheep	LKZ	10	4459	N:28°58′951″<br>E:090°23′757″	KP228955-KP228964	Kexi Village, Langkazi Town, Langkazi County, Shannan Territory of Tibet Autonomous Region
Gangba Tibetan sheep	GB	77	4403	N:28°15′281″<br>E:088°24′787″	KP228670-KP228746	Yulie Village, Gangba Town, Gangba County, Rikaze Territory of Tibet Autonomous Region
Jiangzi Tibetan sheep	JZ	46	4398	N:28°55′113″<br>E:089°47′692″	KP228909-KP228954	Redingvillage, Cheren Town, Jiangzi County, Shannan Territory of Tibet Autonomous Region
Zashijia Tibetan sheep	ZSJ	60	4260	N:34°12′131″<br>E:092°25′463″	KP229238-KP229297	Tongka Village, Zhiqu Town, Zhiduo County, Yushu Autonomous Prefecture, Qinghai Province
Qinghai Oula sheep	QH	43	3630	N:34°16′433″<br>E:101°32′141″	KP229023-KP229065	Jianke Village, Kesheng Town, Henan Mongolian Autonomous County, Qinghai Province
Qilian White Tibetan sheep	QL	46	3540	N:42°20′178″<br>E:116°64′618″	KP229128-KP229173	Qilian Town, Qilian County, Delingha City, Mongolian Autonomous State, Qinghai Province
Tianjun White Tibetan sheep	TJ	64	3217	N:42°18′158″<br>E:116°42′210″	KP229174-KP229237	Shengge Countryside, Tianjun County, Delingha City, Mongolian Autonomous State, Qinghai Province
Guide Black Fur sheep	GD	38	3100	N:38°61′152″<br>E:103°32′160″	KP228747-KP228784	Senduo Town, Guinan County, Hainan Tibetan Autonomous State, Qinghai Province
Gannan Oula sheep	GN	43	3616	N:33°51′312″<br>E:101°52′424″	KP228833-KP228875	Daerqing administrative village, Oula Town, Maqu County, Gannan Tibetan Autonomous State, Gansu Province
Qiaoke Tibetan sheep	QK	62	3410	N:35°42′106″<br>E:102°42′210″	KP229066-KP229127	Waeryi Village, Qihama Town, Maqu County, Gannan Tibetan Autonomous State, Gansu Province
Minxian Black Fur sheep	MX	58	3180	N:36°54′48″<br>E:103°94′107″	KP228965-KP229022	Taizi Village, Qingshui Town, Minxian County, Dingxi City, Gansu Province
Ganjia Tibetan sheep	GJ	48	3022	N:35°32′49″<br>E:102°40′802″	KP228785-KP228832	Xike Village, Ganjia Town, Xiahe County, Gannan Tibetan Autonomous State, Gansu Province

Supplementary Table S2. Mitochondrial genomes of the 19 populations of 237 sequences included in phylogenetic analyses of this study.

Population	Population code	Sample size	Accession no.	Country/location	Reference
Akkaraman sheep	AKA	4	DQ097423-DQ097426	Turkey	Pedrosa S., et al., 2005
Bannur sheep	BN	19	FJ218019-FJ218037	India	Unpublished
Bashibai sheep	BSB	3	DQ903208-DQ903210	China	Wang X., et al., 2006
Cypriot mouflon	CM	5	FR873149-FR873153	Italia	Unpublished
Garole sheep	GA	21	FJ218038-FJ218058	India	Unpublished
Henan Big Tail sheep	HN	3	DQ903213-DQ903215	China	Wang X., et al., 2006
Kolhapuri sheep	KO	24	FJ218059-FJ218082	India	Unpublished
Deccani sheep	DE	29	FJ218083-FJ218111	India	Unpublished
Madgyal sheep	MD	12	FJ218112-FJ218123	India	Unpublished
Morkaraman sheep	MOR	12	DQ097418-DQ097429	Turkey	Pedrosa S., et al., 2005
Bulkhi sheep	BU	49	JX235833-JX235881	Pakistani	Unpublished
Sangamneri sheep	SN	27	FJ218124-FJ218150	India	Unpublished
Snow sheep	SS	5	AJ867261-AJ867265	China	Bunch,T.D., et al., 2006
Tuj sheep	TUJ	8	DQ097407-DQ097414	Turkey	Pedrosa S., et al., 2005
Argali	Argali	11	AJ867266- AJ867276	China	Bunch,T.D., et al., 2006
Musimon	Musimon	2	HM236184-HM236185	Germany	Meadows JRS,et al., 2011
Bos taurus (cattle)	BTC	1	V00654	China	Anderson et al., 1982
Bos mutus (wild yak)	BMWY	1	KR106993	China	Unpublished
Bos grunniens (domestic yak)	BGDY	1	KJ704989	China	Wu et al., 2014

References

Pedrosa, S., Uzun, M., Arranz, J.J., Gutie´rrez-Gi, B., Primitivo F. S., Bayo´n, Y. Evidence of three maternal lineages in near eastern sheep supporting multiple domestication events. *Proc. R. Soc. B.* **2005**, 272, 2211-2217.

WANG, X., MA,Y.H., CHEN, H. Analysis of the Genetic Diversity and the Phylogenetic Evolution of Chinese Sheep Based on Cyt *b* Gene Sequences. *Acta Genetica Sinica*. **2006**, 33(12), 1081-1086.

BUNCH,T.D., WU, C., ZHANG, Y.P., WANG, S. Phylogenetic Analysis of Snow Sheep (Ovis nivicola) and Closely Related Taxa. *Journal of Heredity*. **2006**, 97(1), 21-30.

Meadows, J.R.S., Hiendleder, S., Kijas, J.W. Haplogroup relationships between domestic and wild sheep resolved using a mitogenome panel. *Heredity*. **2011**, 106, 700-706.

Anderson, S., de Bruijn, M.H., Coulson, A.R., Eperon, I.C., Sanger, F., Young, I.G. Complete sequence of bovine mitochondrial DNA. Conserved features of the mammalian mitochondrial genome. *J Mol Biol*. **1982**, 156(4):683-717.

Wu, X., Ding, X., Chu, M., Guo, X., Bao, P., Liang, C., Yan, P. Characterization of the complete mitochondrial genome sequence of Gannan yak (Bos grunniens). *Mitochondrial DNA*. **2016**, 27(2):1014-5.

Supplementary Table S3. Estimates of pairwise F_{ST} values and *p*-value (below the diagonals) and genetic distance (above the diagonals) between and within (diagonal) 15 Tibetan sheep populations.

Population	DM	AW	HB	LKZ	GB	JZ	ZSJ	QH	QL	TJ	GD	GN	QK	MX	GJ
DM	**0.020**	0.013	0.021	0.020	0.021	0.029	0.033	0.024	0.018	0.016	0.022	0.025	0.021	0.017	0.018
AW	0.020<br>0.056	**0.004**	0.015	0.015	0.016	0.027	0.030	0.018	0.013	0.010	0.017	0.022	0.016	0.009	0.012
HB	0.013<br>0.046	0.039<br>0.031	**0.024**	0.023	0.024	0.032	0.036	0.027	0.021	0.019	0.024	0.028	0.024	0.019	0.021
LKZ	0.000<br>0.014	0.015<br>0.045	0.000<br>0.031	**0.024**	0.023	0.023	0.036	0.026	0.021	0.018	0.024	0.020	0.023	0.018	0.020
GB	0.024<br>0.031	0.044<br>0.036	0.000<br>0.049	0.009<br>0.032	**0.024**	0.032	0.036	0.027	0.022	0.019	0.025	0.028	0.024	0.020	0.021
JZ	0.025<br>0.071	0.045<br>0.037	0.004<br>0.024	0.005<br>0.031	0.003<br>0.020	**0.038**	0.043	0.035	0.030	0.030	0.032	0.035	0.032	0.031	0.031
ZSJ	0.071<br>0.010	0.118<br>0.052	0.069<br>0.053	0.038<br>0.034	0.070<br>0.037	0.066<br>0.056	**0.048**	0.039	0.034	0.032	0.037	0.040	0.036	0.025	0.034
QH	0.034<br>0.037	0.044<br>0.036	0.009<br>0.019	0.006<br>0.014	0.006<br>0.045	0.012<br>0.018	0.031<br>0.050	**0.030**	0.024	0.022	0.028	0.031	0.025	0.023	0.023
QL	0.009<br>0.043	0.043<br>0.046	0.000<br>0.046	0.010<br>0.043	0.008<br>0.043	0.019<br>0.014	0.063<br>0.041	0.031<br>0.056	**0.020**	0.017	0.022	0.026	0.022	0.022	0.018
TJ	0.017<br>0.041	0.043<br>0.047	0.008<br>0.019	0.012<br>0.041	0.008<br>0.044	0.027<br>0.000	0.114<br>0.000	0.026<br>0.033	0.003<br>0.049	**0.014**	0.020	0.025	0.020	0.022	0.015
GD	0.010<br>0.031	0.042<br>0.029	0.000<br>0.038	0.002<br>0.058	0.002<br>0.047	0.003<br>0.031	0.091<br>0.068	0.019<br>0.072	0.000<br>0.055	0.006<br>0.030	**0.026**	0.029	0.025	0.020	0.022
GN	0.017<br>0.034	0.041<br>0.037	0.000<br>0.041	0.000<br>0.039	0.000<br>0.043	0.001<br>0.056	0.070<br>0.039	0.006<br>0.033	0.005<br>0.033	0.006<br>0.000	0.000<br>0.036	**0.032**	0.029	0.025	0.026
QK	0.021<br>0.045	0.043<br>0.027	0.000<br>0.042	0.008<br>0.021	0.000<br>0.059	0.004<br>0.000	0.078<br>0.024	0.009<br>0.030	0.003<br>0.048	0.006<br>0.024	0.000<br>0.033	0.000<br>0.045	**0.025**	0.023	0.021
MX	0.035<br>0.020	0.049<br>0.031	0.022<br>0.012	0.021<br>0.030	0.027<br>0.000	0.038<br>0.000	0.080<br>0.000	0.027<br>0.000	0.036<br>0.012	0.034<br>0.000	0.020<br>0.009	0.022<br>0.000	0.026<br>0.000	**0.012**	0.015
GJ	0.025<br>0.051	0.044<br>0.058	0.005<br>0.020	0.009<br>0.020	0.003<br>0.032	0.015<br>0.000	0.071<br>0.012	0.013<br>0.040	0.014<br>0.027	0.010<br>0.000	0.010<br>0.035	0.004<br>0.020	0.004<br>0.024	0.030<br>0.000	**0.017**

F_{ST} = Wright's subpopulation within total population F-statistic. Below the diagonals of upper row is estimates of pairwise F_{ST} values and lower row is *p*-value.
